# Preoperative monocyte‐to‐HDL‐cholesterol ratio predicts early recurrence after radiofrequency maze procedure of valvular atrial fibrillation

**DOI:** 10.1002/jcla.23595

**Published:** 2020-09-25

**Authors:** Ailiya Adili, Yali Wang, Xiyu Zhu, Hailong Cao, Fudong Fan, Xinlong Tang, Qing Zhou, Dongjin Wang

**Affiliations:** ^1^ Department of Cardio‐Thoracic Surgery Nanjing Drum Tower Hospita,the Affiliated Hospital of Nanjing University Medical School Nanjing China

**Keywords:** early recurrence, monocyte‐to‐high‐density lipoprotein ratio, radiofrequency maze procedure, valvular atrial fibrillation

## Abstract

**Background:**

Monocyte‐to‐high‐density lipoprotein (M/H) ratio has emerged as a novel cardiovascular prognostic biomarker. We aimed to evaluate the prognostic values of M/H with early recurrence in persistent valvular atrial fibrillation (AF) patients after radiofrequency (RF) maze procedure.

**Methods:**

We retrospectively analyzed 131 consecutive persistent AF patients with valvular heart diseases who were followed up 3 months after RF maze procedure. Their clinical data were recorded. Logistic regression analyses were performed for significant predictors. Receiver operating characteristic analysis was used for validation with corresponding area under the curve.

**Results:**

70 (53.4%) patients experienced early recurrence after procedure. Patients with early recurrence were older, have longer AF duration history, larger left atria diameter (LAD), higher plasma C‐reactive protein (CRP), lower triglycerides (TG), lower cholesterol (TC), increased monocyte counts, lower HDL cholesterol, and increased M/H ratio. In multivariate analysis, age (OR 1.1 95% CI 1.0‐1.1 *P* = .003), LAD (OR 2.1, 95%CI 1.2‐3.5, *P* = .006), TG (OR 0.35, 95% CI 0.15‐0.84, *P* = .019), M/H (OR 6.1, 95% CI 2.9‐13.0, *P* < .001) were significantly independent predictors of AF early recurrence. M/H ratio demonstrated a significant predictive value (AUC = 0.77, sensitivity 89.0%, specificity 54%). Further, there was a positive correlation of M/H ratio with CRP and white blood cell.

**Conclusion:**

Preoperative M/H ratio was an independent risk factor of AF early recurrence following RF maze operation. M/H ratio should be considered in prediction of early recurrence for valvular AF patients.

## Graphical abstract

Main Line.

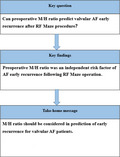

## 1 INTRODUCTION

Atrial fibrillation is the most commonly encountered cardiac arrhythmia in clinical practice, contributing to increased risks of cardiovascular events and adversely affects quality of life.^1^ Maze IV procedure based on radiofrequency ablation is widely accepted as an effective treatment for valvular AF.^2^, ^3^ Studies suggest that surgical ablation (known as Maze procedure) is superior to drug therapy by increasing the rate of freedom from AF among patients with persistent or long‐standing persistent AF.^2^, ^3^, ^4^, ^5^ However, the recurrence of AF is common and mostly occurring in the first 3 months after ablation (defined as early recurrence).^6^, ^7^ During the first 3 months after surgery, half or more of patients experience atrial arrhythmias.^8^ In recent decades, much attention has been dedicated to the clinical importance of early recurrence as it is found to be a powerful independent predictor of late recurrence.^9^, ^10^, ^11^ Early restoration of sinus rhythm following ablation deceases adverse atrial remodeling and improves the long‐term outcomes.^9^ Therefore, it is of great significance to search for preoperative biomarker for early recurrence to avoid procedure‐related risks and take early intervention as soon as possible. At the meantime, it is helpful to practice individual therapy to maximize clinical efficacy and to provide insight into more accurate therapies.

Inflammation and oxidative stress are shown to be significant contributors of AF structural remodeling process.^12^, ^13^ Various inflammatory biomarkers including CRP, IL‐1, IL‐6, IL‐8, and TNF have been widely studied and demonstrated to be closely associated with electrical and structural atrial remodeling and thrombogenesis in AF.^12^ Furthermore, there is a large amount of data showing that oxidative stress has an important role in development of AF.^13^, ^14^

Recently, studies suggested that the M/H ratio, which was strongly associated with inflammation and oxidative stress, has emerged as a novel and widely available cardiovascular prognostic biomarker.^15^ Canpolat et al evaluated the late recurrence prognostic value of M/H ratio among non‐valvular AF patients after catheter ablation.^16^ However, its prognostic value of valvular AF early recurrence at 3 months after RF maze procedure remains poorly studied. Thus, we aimed to investigate the prognostic value of M/H for valvular AF early recurrence at 3 months after RF maze procedure in patients undergoing mitral‐valve surgery.

## 2 METHODS

### 2.1 Study population

In this study, 150 consecutive persistent AF patients who required RF maze procedure concomitant mitral‐valve surgery were enrolled. All patients provided written informed consent admitted to the Affiliated Drum Tower Hospital of Nanjing University Medical School between September 2018 and June 2019. A pre‐ablation 7‐day Holter and comprehensive transthoracic echocardiographic examination were performed to establish that all patients had persistent valvular AF. Persistent AF was defined as any AF episode lasting longer than 7 days or requiring termination by cardioversion. The study was in compliance with the principles outlined in the Declaration of Helsinki Declaration and approved by the institutional review board of Nanjing Drum Tower Hospital (IRB number 2016‐151‐01).

Clinical data were collected. Nineteen patients were excluded: 1 case died in hospital; 1 case died of sudden death after discharge; 2 cases died of heart failure after discharge; 3 cases received pacemaker implantation because of long RR intervals or advanced atrioventricular block. Patients who had uncontrolled thyroid dysfunction, pregnancy and subjects with recent infection, malignancies, blood dyscrasias, auto‐immune diseases, renal failure, or hepatic failure, current therapy with corticosteroids and non‐steroidal anti‐inflammatory drugs were excluded from the study. And another six cases were lost at follow‐up. Finally, a total of 131 patients eventually achieved complete clinical data and follow‐ups.

### 2.2 Data collection

Baseline characteristics were collected upon admission, including age, sex, smoking and drinking history, body mass index (BMI), medical history, drug use, electrocardiogram (ECG), and transthoracic echocardiography (TTE). Blood samples were collected the first morning of hospitalization and were taken as fasting blood samples to measure preoperative routine blood analyses and a panel of biochemistry markers, including lipid parameters.

### 2.3 Follow‐up

Heart rhythm was continuously monitored after surgery. Dual‐chamber stimulation at 80 bpm through epicardial temporary pacing wires was used in most of patients for the first 48 hours post‐operation to avoid severe brady arrhythmias. Early recurrence was defined as any episode of AF, atrial flutter or atrial tachycardia that lasted greater than 30s in the first 3 month after surgical ablation.^7^ Repeat ablation or electronic cardioversion at follow‐up time was classified as a recurrence.^7^ Patients had scheduled clinical visits. 24‐hour Holter monitoring was routinely performed in all patients in the first 3 months after surgery. Moreover, patients would receive electrocardiography monitoring in local clinics at any time if they had AF‐related symptoms.

### 2.4 Statistical analysis

All statistical analysis was conducted using SPSS version 22.0 (IBM). Values were presented as median (interquartile range, IQR) and mean ± SD for continuous variables, and proportions for categorical variables. For the comparison between the two groups, Student's *t* test (normally distributed) or Mann‐Whitney test (non‐normally distributed) was used for continuous variables, and χ^2^ test was utilized for categorical variables. Multivariate logistic regression analysis, which included variables with a *P* < .05 found on univariate analysis, was performed to identify the predictors of early recurrence. All odds ratios (OR) were given with the 95% confidence interval (CI). A receiver operating characteristic (ROC) curve was used to test the ability of prediction model and the area under the curve (AUC) determined the predictive value. Sensitivity and Specificity were investigated by the Fisher's exact test. The associations between the continuous variables were assessed using a Pearson or Spearman rank correlation test. *P* < .05 was considered statistically significant, and all statistical tests were two‐sided.

## 3 RESULTS

A total of 131 were enrolled in this study. Follow‐up was completed in all patients after surgery. The clinical, echocardiographic, and laboratory characteristics of patients with or without early recurrence were comparable reported in Table 1.

**TABLE 1:** Baseline characteristics of the study population

Parameters	Total N = 131	Recurrence (−) N = 61	Recurrence (+) N = 70	*P*
Age (y)	60 (54‐67)	56 (52‐66)	62 (55‐68)	.016*
Male sex	54 (42.2)	28 (45.9)	26 (42.6)	.310
BMI (kg/m^2^)	23.5 (21.0‐25.7)	23.5 (20.9‐25.6)	23.7 (20.9‐25.8)	.654
Duration of AF history (month)	24 (3‐120)	12 (1.5‐78)	36 (12‐120)	.002*
Hypertension	44 (33.6)	22 (36.1)	22 (31.4)	.575
Diabetes mellitus	9 (6.9)	5 (8.2)	4 (5.7)	.575
Coronary artery disease	26 (19.8)	14 (23)	12 (17.1)	.453
Chronic heart failure	45 (34.4)	17 (27.9)	28 (40)	.128
Previous stroke/TIA	15 (11.5)	7 (11.5)	8 (11.4)	.993
Current smoker	11 (8.4)	7 (11.5)	4 (5.7)	.236
Alcohol intake	7 (5.3)	5 (8.2)	2 (2.9)	.175
LAD (cm)	5.2 (4.7‐6.0)	5 (4.5‐5.4)	5.3 (4.9‐6.3)	.005*
LVEF (%)	53 (47‐58)	55 (50‐57)	51 (44‐58)	.111
Preoperative NYHA, n (%)				
Ⅰ	4 (3.1)	4 (6.6)	0 (0)	.144
Ⅱ	51 (38.9)	25 (41)	26 (37.1)	
Ⅲ	73 (55.7)	31 (50.8)	42 (60)	
Ⅳ	3 (2.3)	1 (1.6)	2 (2.9)	
ACEI/ARB	37 (28.2)	17 (27.9)	20 (28.6)	.929
Antiarrhythmic drug	58 (44.3)	26 (42.6)	32 (45.7)	.722
Statins	26 (19.8)	14 (23)	12 (17.1)	.406
CHA2DS2‐VASc	2 (2‐3)	2 (2‐3)	2 (2‐3)	.856
WBC (10^9^/L)	5.6 (4.6‐6.6)	5.7 (4.9‐6.6)	5.4 (4.3‐6.7)	.678
Neutrophil (10^9^/L)	3.1 (2.4‐3.8)	3.3 (2.5‐3.9)	3.0 (2.3‐3.8)	.613
Monocyte (10^9^/L)	0.4 (0.3‐0.5)	0.4 (0.3‐0.5)	0.5 (0.4‐0.6)	<.001*
BNP (pg/ml)	231 (145‐418)	268 (144‐577)	214 (148‐383)	.511
CRP (mg/L)	4 (2.7‐5.2)	3.3 (2.3‐4.6)	4.7 (3.2‐6.2)	<.001*
Triglycerides (mmol/L)	1.1 (0.8‐1.5)	1.2 (0.9‐1.6)	1.0 (0.8‐1.3)	.011*
Cholesterol (mmol/L)	3.6 (3.1‐4.1)	3.7 (3.4‐4.3)	3.5 (2.8‐4.0)	.016*
HDL (mmol/L)	1.0 (0.8‐1.2)	1.0 (0.9‐1.2)	1.0 (0.8‐1.1)	.003*
LDL (mmol/L)	2.0 (1.7‐2.5)	2.1 (1.8‐2.6)	1.9 (1.6‐2.4)	.133
Serum creatinine (µmol/L)	69 (59‐80)	69 (59‐78)	68 (57‐82)	.947
M/H	0.4 (0.3‐0.6)	0.3 (0.2‐0.5)	0.5 (0.4‐0.7)	<.001*

Note

Continuous variables are presented as median (interquartile range, IQR) and mean ± SD, while categorical variables are presented as patients (%). Student's *t* test (normally distributed) or Mann‐Whitney test (non‐normally distributed) was used for continuous variables, and χ^2^ test was utilized for categorical variables.

Abbreviations: LAD, left atrial diameter; LVEF, left ventricular ejection function; ACEI, angiotensin‐converting enzyme inhibitors; ; ARB, angiotensin receptor blockers; BMI, body mass index; BNP, B‐type natriuretic peptide; CRP, C‐reactive protein; HDL, high‐density lipoprotein cholesterol; LDL, low‐density lipoprotein cholesterol; M/H, monocyte‐to‐HDL level ratios; NYHA, New York Heart Association; WBC, white blood cell.

* Statistically significant value (*P* < .05).

**FIGURE 1:**
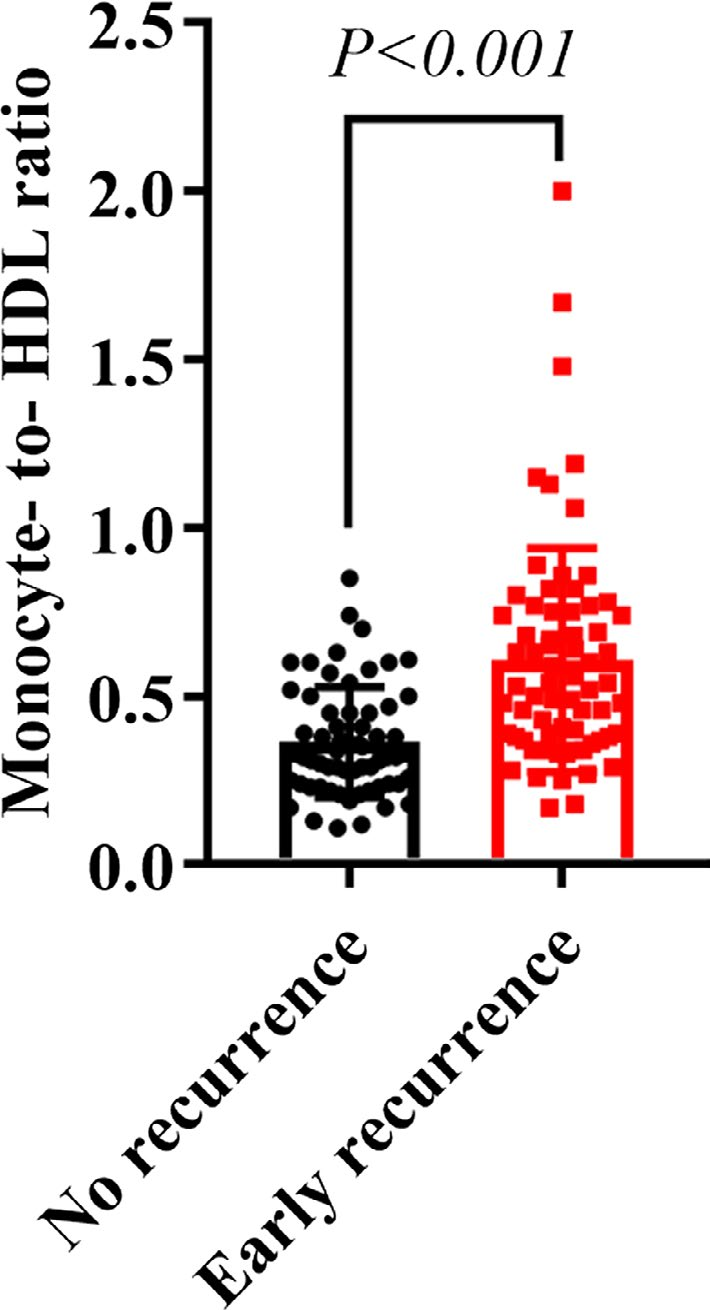
Pre‐ablation monocyte‐to‐HDL ratio (M/H ratio) in patients with early recurrence compared with those without early recurrence (*P* < .001)

70/131 (53.4%) patients experienced early recurrence after procedure. Those who were diagnosed early recurrence were older (*P* = .016), have longer AF duration history (*P* = .002), larger LAD (*P* = .005), higher CRP (*P* < .001), lower TG (*P* = .011), lower TC (*P* = .016), increased monocyte counts (*P* < .001), lower HDL cholesterol (*P* = .003), and increased M/H ratio (*P* < .001) comparing with those without early recurrence (Figure 1).

Clinical, echocardiographic, and laboratory data were tested to predict early recurrence using the logistic regression univariate and multivariate analysis (Table 2).

**TABLE 2:** Univariate and multivariate analysis of risk factors for early recurrence after radiofrequency maze procedure

Variables	Univariate model			Multivariate model		
	OR	95% CI	P	OR	95% CI	P
Age	1.0	1.0‐1.1	0.017*	1.1	1.0‐1.1	.003*
BMI	1.0	0.9‐1.1	0.686			
Duration of AF history	1.01	1.00‐1.01	0.015*	1.00	1.00‐1.01	.287
LAD	1.90	1.3‐2.9	0.002*	2.1	1.2‐3.5	.006*
LVEF	1.0	0.9‐1.0	0.046*	1.0	0.9‐1.1	.563
BNP	1.00	1.00‐1.01	0.646			
CRP	1.3	1.1‐1.6	0.002*	1.2	1.0‐1.5	.092
Triglycerides	0.43	0.21‐0.86	0.018*	0.35	0.15‐0.84	.019*
Cholesterol	0.60	0.39‐0.95	0.027*	1.0	0.5‐2.0	.908
M/H	4.5	2.3‐8.6	<0.001*	6.1	2.9‐13.0	<.001*

Abbreviations: BMI, body mass index; BNP, B‐type natriuretic peptide; CI, confidence interval; CRP, C‐reactive protein; LAD, left atrial diameter; LVEF, left ventricular ejection function; M/H, monocyte‐to‐HDL level ratios; OR, odds ratio.

* Statistically significant value (*P* < .05).

Univariate analysis demonstrated that age (*P* = .017), duration of AF history (*P* = .015), LAD (*P* = .002), LVEF (*P* = .046), CRP (*P* = .002), TG (*P* = .018), TC (*P* = .027), and M/H (*P* < .001) were associated with early recurrence. In multivariate analysis, age (OR 1.1 95% CI 1.0‐1.1 *P* = .003), LAD (OR 2.1, 95% CI 1.2‐3.5, *P* = .006), TG (OR 0.35, 95% CI 0.15‐0.84, *P* = .019), M/H (OR 6.1, 95% CI 2.9‐13.0, *P* < .001) were significantly independent predictors of AF early recurrence (Table 2).

**FIGURE 2:**
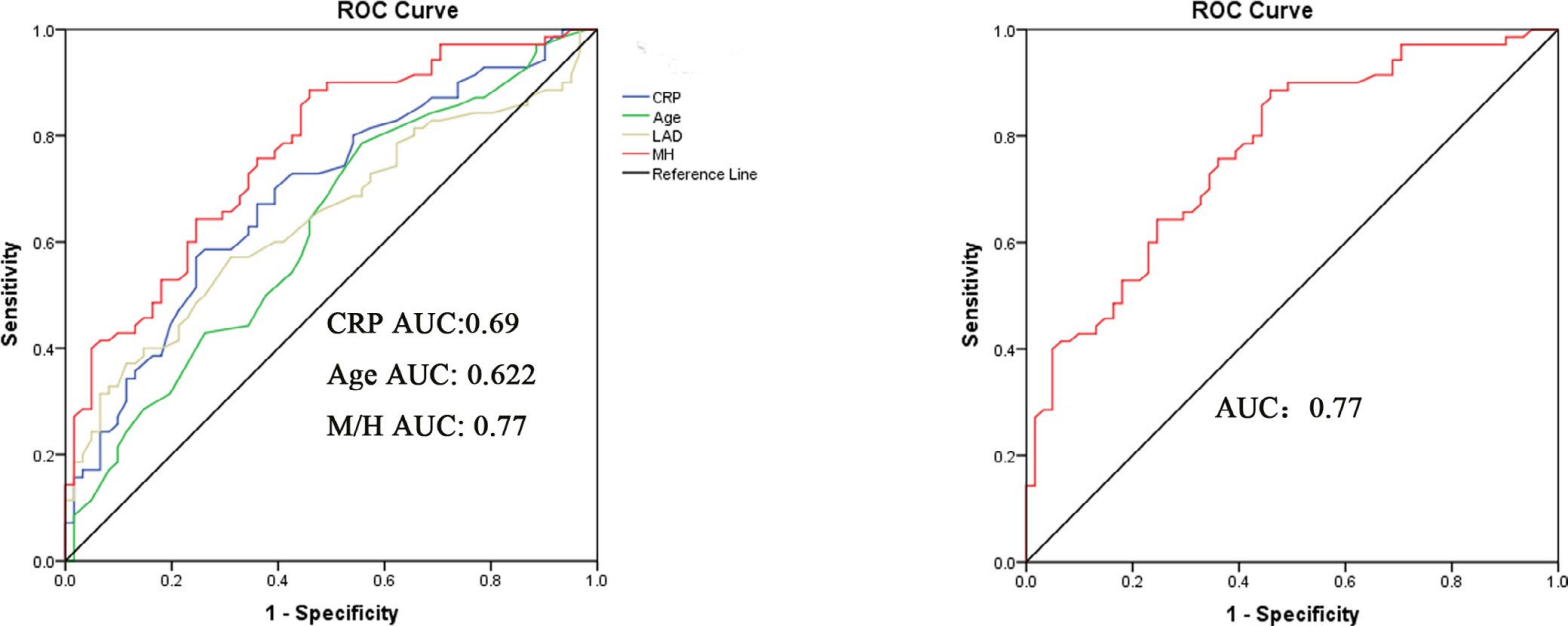
Receiver operating characteristic curve (ROC) of pre‐operative risk factors for predicting AF early recurrence after surgery

Further, ROC analysis was used to evaluate the relation between pre‐ablation M/H ratio and AF recurrence. M/H ratio demonstrated a significant predictive value with AUC of 0.77 (95% CI 0.69‐0.85, *P* < .001). LAD, CRP, and Age showed a predictive value with AUC of 0.644 (95% CI 0.55‐0.738, *P* = .005), 0.69 (95% CI 0.599‐0.78, *P* < .001), 0.622 (95% CI 0.526‐0.718, *P* = .016), respectively (Figure 2). Moreover, the optimal cut‐off value was 0.33 for M/H. The corresponding sensitivity and specificity were 89% and 54% by M/H.

Stratified by the cut‐off value, association of M/H with several clinical characteristics was evaluated. Elevated M/H was significantly associated with BMI (*P* = .028), LAD (*P* = .02), white blood cell (WBC) (*P* = .004), CRP (*P* = .003), BNP (*P* = .025), Cholesterol level (*P* = .003), and AF recurrence (*P* < .001) (Table 3).

**TABLE 3:** Clinical characteristics in AF patients according to different M/H levels

Parameters	M/H ≥ 0.33 n = 93	M/H < 0.33 n = 38	P
Age (y)	59 (53‐67)	61 (54‐67)	.501
Male sex	41 (44)	13 (34.2)	.297
BMI (kg/m^2^)	23.8 (21.5‐25.9)	22.4 (19.9‐24.0)	.028
Duration of AF history (month)	24 (6‐120)	24 (2‐102)	.452
Hypertension	28 (30.1)	16 (42.1)	.187
Diabetes mellitus	6 (6.5)	3 (7.9)	.767
Coronary artery disease	19 (20.4)	7 (18.4)	.794
Chronic heart failure	32 (34.4)	13 (34.2)	.95
Previous Stroke/TIA	10 (10.8)	5 (13.2)	.695
Current smoker	6 (6.5)	5 (13.2)	.209
Alcohol intake	3 (3.2)	4 (10.5)	.092
LAD (cm)	5.3 (4.8‐6.2)	5.0 (4.5‐5.3)	.02
LVEF (%)	52 (45‐57)	54 (48‐58)	.215
Preoperative NYHA n (%)			
I	2 (2.2)	2 (5.3)	.388
II	33 (35.5)	18 (47.4)	
III	56 (60.2)	17 (44.7)	
IV	2 (2.2)	1 (2.6)	
WBC	5.8 (4.8‐6.8)	5.0 (4.2‐5.8)	.004
Neutrophil (10^9^/L)	3.2 (2.6‐4.0)	3.4 (2.4‐3.4)	.113
CRP	4.4 (3.1‐5.7)	2.9 (2.2‐4.5)	.003
BNP	249 (169‐522)	162 (110‐364)	.025
Triglycerides	1.1 (0.8‐1.5)	1.0 (0.8‐1.5)	.644
Cholesterol	3.5 (3.0‐4.0)	3.8 (3.5‐4.5)	.003
LDL	1.9 (1.6‐2.4)	2.2 (1.8‐2.7)	.079
Serum creatinine	71 (59‐82)	63 (56‐76)	.068
AF recurrence (+)	63 (67.8)	7 (18.4)	<.001

Note

Continuous variables are presented as median (interquartile range, IQR) and mean ± SD, while categorical variables are presented as patients (%). Student's *t* test (normally distributed) or Mann‐Whitney test (non‐normally distributed) was used for continuous variables, and χ^2^ test was utilized for categorical variables.

Abbreviations: AF, atrial fibrillation; BMI, body mass index; BNP, B‐type natriuretic peptide; CRP, C‐Reactive Protein; LAD, left atrial diameter; LDL, low‐density lipoprotein cholesterol; LVEF, left ventricular ejection function; M/H, monocyte‐to‐HDL ratio; NYHA, New York Heart Association; WBC, white blood cell.

* Statistically significant value (*P* < .05).

**FIGURE 3:**
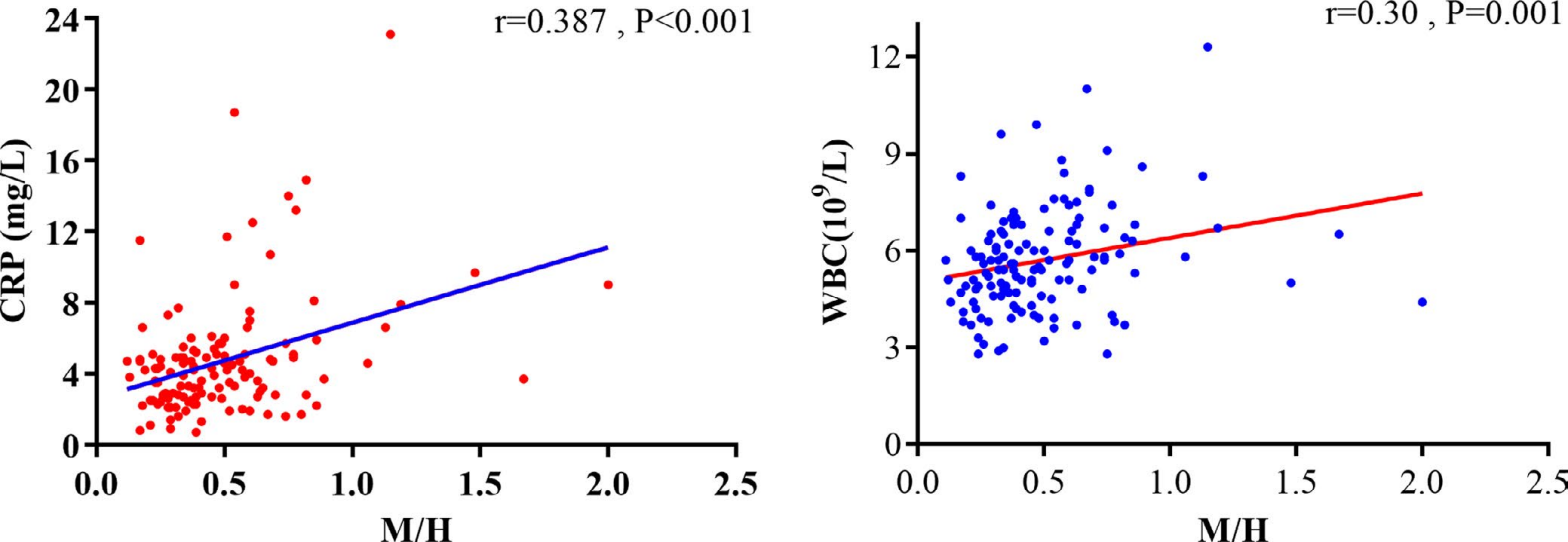
Correlation of the preoperative monocyte‐to‐HDL ratio (M/H ratio) with CRP and WBC

In addition, correlation analysis revealed that there was a positive correlation of M/H ratio with CRP (*r* = 0.387, *P* < .001) and WBC (*r* = 0.3, *P* = .001) (Figure 3).

## 4 DISCUSSION

In this retrospective study, we analyzed the prognostic value of inflammation and oxidative stress‐based marker M/H ratio among 131 valvular AF patients who underwent RF maze procedure concomitant mitral‐valve surgery. We demonstrated that 70 patients (53.4%) experienced early recurrence after surgery during follow‐up time. Results showed that M/H ratio is a strong and independent predictor of AF early recurrence. M/H ratio demonstrated a significant predictive value with AUC of 0.77 (95% CI 0.69‐0.85, *P* < .001). Moreover, the optimal cut‐off value was 0.33 and corresponding sensitivity and specificity were 89%, 54% by M/H. We also found that M/H ratio has positive correlation with CRP and WBC in AF patients.

Therapy of persistent valvular AF has high failure rates, with 20%‐60% of patients showing recurrence of AF within three months after ablation.^17^ Herein, at a mean follow‐up of 3 months, we found high recurrence rate of AF (53. 4%).One reason is the clinical characteristics of our patients. There were 33 patients (25.2%) found preoperative large left atrium (>60 mm) in our study.^3^ Most of them are older and experienced longer AF duration. Another reason might be our surgical technique. For large left atrium, the maze around the pulmonary veins and mitral annulus might be not narrow enough to interrupt reentrant circuits.

Recently, some studies have reported the importance of early recurrence as it is associated with late recurrence, and even is the strongest predictor of late recurrence.^9^, ^10^, ^11^ Early intervention for early recurrent AF is associated with excellent long‐term outcome.^18^ However, there are limited biomarkers available of early recurrence occurred in 3 months in AF patients after RF maze procedure. In present investigation, we showed that M/H level was identified as AF early recurrence marker, and our study find that it has important prognostic value.

Inflammation and oxidative stress, as major contributors to cardiovascular disease, have been implicated as risk factors for AF.^12^, ^13^, ^14^ Mediators of the inflammatory response are closely related to electrical and structural remodeling in atria, thereby leading to increased vulnerability to AF.^19^ Inflammation also has an effect on calcium homeostasis and connexins, which are associated with triggers of AF and heterogeneous atrial conduction. The activation of fibrotic pathways is also mediated by inflammatory pathways, which can all contribute to structural remodeling of the atria.^12^ Experimental and clinical studies have shown that oxidative stress is closely associated with AF.^13^, ^14^, ^20^ Oxidative stress cross‐linked with inflammation and consequently result in atrial fibrosis which may play a pivotal role in substrate modification by electrical and structural remodeling of AF pathophysiology and increase the susceptibility for AF.^14^

Monocytes, as the most important sources of pro‐inflammatory and pro‐oxidant cytokines, play a key role in the initiation, perpetuation, and recurrence of AF by triggering an inflammatory cascade.^12^, ^21^ Monocyte/macrophage was found to infiltrate the atrial myocardium in patients with AF with an underlying structural heart disease which leads to detrimental effects on AF pathogenesis.^22^, ^23^

Dyslipidemia, especially high TC, TG, LDL levels, and low HDL levels, is well‐established risk factors for cardiovascular diseases.^24^ However, the association between lipid profile and AF recurrence is inconsistent.^25^, ^26^ The inconsistent results may be attribute to various factors, such as geographical and ethnic variations, other combined cardiovascular risk factors, follow‐up duration, and thrombosis state. Recently, the relationship between high‐density lipoprotein cholesterol (HDL) and cardiovascular disease has been widely investigated. Low HDL has been associated with increased levels of inflammation.^27^, ^28^ Studies showed that alterations in HDL function may associate with AF initiation or progression and HDL can be useful biomarker to predict the occurrence of AF after surgery.^29^, ^30^ It can be suggested that low HDL leads to detrimental effects on AF pathogenesis by reducing its anti‐inflammatory and antioxidant effects.

In recent study, M/H has emerged as a new and valuable biomarker in cardiovascular disease, providing important prognostic information. A significant prognostic value of M/H in predicting post ablation recurrences of AF and other cardiovascular disease outcome have been reported recently.^15^, ^16^, ^31^, ^32^ However, valvular AF was excluded from their study.^16^ Valvular AF may persist sustained chronic inflammation, ultimately leading to ECM remodeling.^33^ Additionally, M/H prognostic value of valvular AF early recurrence at 3 months after RF maze procedure remains poorly studied. In accordance with previous studies, we found that preoperative higher M/H levels were independently associated with a significantly increased risk of AF early recurrence following the RF maze operation. Moreover, we also find that M/H ratio was significantly associated with BMI, LAD, WBC, CRP, BNP, and TC level. M/H ratio was positively correlated with CRP and WBC which are relatively biomarker of systemic inflammation in body and predict of all‐cause cardiovascular mortality.^34^, ^35^, ^36^

In conjunction with the literature, our results supported that combined clinical parameters which can reflect inflammation and oxidative stress have an essential role in the development of AF and have valuable prognostic value. A deep understanding of the biomarker underlying different clinical scenarios in AF is necessary for the development of specific therapeutic strategies for the primary or secondary prevention of this arrhythmia. At the meanwhile, it can help with clinicians choose more accurate therapies or early intervention for AF patients.

### 4.1 Limitations

Of course, the current study has several limitations. First, AF recurrences can be asymptomatic and undetectable. We may underestimate the incidence of early recurrence. Second, M/H ratio may only reflect one of the factors rather than reflecting the whole mechanisms for structural remodeling. Thus, it might not be generalized to all AF populations and should be cautious to compare the recurrence rate between different ablation strategies. Repeated and multiple measurements of M/H with large population may help us better evaluate and confirm our findings. Third, whether preoperative intervention on M/H ratio can reduce the AF recurrence for specific patients were not assessed and need to be further investigation.

## 5 CONCLUSIONS

Preoperative M/H ratio was an independent risk factor of AF early recurrence following RF maze operation. M/H ratio should be considered in prediction of early recurrence for valvular AF patients.

## AUTHOR CONTRIBUTIONS

Qing Zhou involved in conception and design. Dongjin Wang involved in administrative support. Hailong Cao and Fudong Fan involved in provision of study materials or patients. Yali Wang and Xiyu Zhu involved in collection and assembly of data. Ailiya Adili involved in data analysis and interpretation. All authors involved in manuscript writing and final approval of manuscript.

## COMPETING INTERESTS

The authors report no proprietary or commercial interest in any product mentioned or concept discussed in this article.

## ETHICS APPROVAL AND CONSENT TO PARTICIPATE

The current study was approved by the institutional review board of Nanjing Drum Tower Hospital (IRB number 2016‐151‐01). The requirement to obtain informed consent from the patient was waived.
